# Supplementary material for: Are carbon emissions trading and green financial instruments synergistic? -Comprehensive quantitative research based on content analysis
Source: PLoS One. 2024 Mar 7;19(3):e0298601. doi: 10.1371/journal.pone.0298601 (PMC10919601; doi:10.1371/journal.pone.0298601)
Supplement: S1 Data — (ZIP) [file pone.0298601.s001.zip › Data/地级市Z_F工作报告.zip!/╦─┤¿/─╧│Σ╩╨/└┤╫╘╣1⁄2╓┌║┼í╛┬φ┐╦╩2╛▌═°í┐_2018.docx]

各位代表：

　　现在，我代表市人民政府，向大会报告工作，请予审议，并请市政协委员提出意见。

一、2017年工作回顾

　　过去一年，是南充发展昂扬奋进、勇开新局的一年。一年来，我们坚持以习近平新时代中国特色社会主义思想为指导，在省委、省政府和市委的坚强领导下，在市人大、市政协的监督支持下，牢牢把握稳中求进工作总基调，紧紧围绕“南充新未来·成渝第二城”战略目标，大力实施“155发展战略”，打好打赢“三场攻坚战”“三场保卫战”“三场持久战”，全面完成各项目标任务，经济社会发展取得全新成绩。全市实现地区生产总值1827.9亿元、增长8.5%，增速居全省第6位、同比上升6位；全社会固定资产投资1820亿元、增长16.6%，增速居全省第3位、同比上升7位；一般公共预算收入103.3亿元、增长14.5%，增速居全省第1位、同比上升3位；社会消费品零售总额888.3亿元、增长13.1%，增速居全省第3位、同比上升8位；城镇居民人均可支配收入28333元、增长9%，增速居全省第3位、同比上升3位；农村居民人均可支配收入12389元、增长9.9%，增速居全省第4位、同比上升5位；主要经济指标增速全面进入全省“一方阵”、排位创历史新高，全市经济呈现出稳中快进的强劲态势，领跑川东北的优势更加明显。

　　一年来，我们主要完成了以下工作：

　　（一）狠抓产业培育、集群发展，转型升级迈出新步伐。 坚持产业兴市、工业强市，加快发展“五大千亿产业集群”，全市产业规模不断壮大、层次不断提升。汽车汽配产业加快突破。实现产值602.8亿元、增长18.9%。吉利一期投产见效、销售整车6200余台，博萨汽配、中国重汽等项目加快建设，吉利二期、重庆银翔等项目成功落户。油气化工产业优化提升。实现产值280.2亿元、增长14.8%。联成化学、石达化工、中石化LNG等项目竣工投产，晟达PTA项目完成中交。丝纺服装产业振兴提速。实现产值397.9亿元、增长11.2%。嘉美印染、依格尔等企业加快技改扩能，“中国网库”电商平台投入运营。成功申报高档丝绸标志等品牌17个。现代物流产业持续壮大。实现产值390亿元、增长16%。南充公共保税仓基本建成，进口产品体验馆开馆运营，川东粮油储备调控中心一期主体完工，中国物流南充分中心、友信龙冷链物流中心等项目加快实施。现代农业蓬勃发展。实现产值925.6亿元、增长10.7%。粮食总产量连续15年居全省首位。建成现代循环农业园区15个、新业态示范样板20个，培育产值过亿元龙头企业16家。认证“三品一标”农产品48个，有机农产品基地达30.2万亩、稳居西部前列。升钟灌区二期、营山金鸡沟水库加快建设，全市新增有效灌面43.5万亩、高标准农田35.6万亩。

　　同时，电子信息、新材料、生物医药等战略性新兴产业实现产值257.8亿元、增长28.8%，三环四期五期、鑫达一期全面达产，太极制药、香雪制药等企业加快发展。全市新增规上工业企业70户，规上工业增加值增长10.2%；服务业增加值增长10.5%。

　　（二）狠抓项目建设、攻坚突破，发展后劲得到新提升。坚持以项目统揽全局工作、组织经济工作，大力实施“五大板块重大工程项目”，222个市级重大项目完成投资1164.3亿元、增长57.3%。突出抓好城市建设项目。金融大道、北部新城一路竣工通车，滨江大道、江东大道、西华路等城市干道提升改造全面完成，高铁商务区、上中坝“印象嘉陵江”等项目加快推进，中心城区实现了多年以来最大范围的城市修复、最大体量的城市改造、最大规模的城建会战。突出抓好交通枢纽项目。蓉京高铁成南达段、汉巴南铁路前期工作进展顺利，全年新增开动车组8对。绵南、营达高速公路和定升、顺蓬营等干线公路加快建设。高坪机场航站楼改扩建工程主体完工，航线增至16条。嘉陵江航运配套工程扎实推进。突出抓好教科文卫项目。新（改、扩）建中小学、幼儿园校舍63.4万平方米，建成标准中心校24所、农村义务教育寄宿制学校42所。中科院南充育成中心、三环研究院、西南交大汽车研究院成功落户，鑫达研究院完成组建，欧香智慧小镇建设加速推进。营山奥体中心、西充体育馆等项目全面竣工，市博物馆、市文化馆等项目加快实施。11个县级医疗机构能力提升项目开工建设。突出抓好旅游三产项目。阆中水城、蓬安相如湖、嘉陵天乐谷等项目加快建设，南部八尔湖成功开园，新增3A级旅游景区6个，实现旅游总收入458.7亿元、增长22.3%。新增国家级电商进农村综合示范县4个，当当网全国农村电商总部基地投入运营。川东北金融中心建设加快推进，全市新增贷款总量居全省第2位。突出抓好产业园区项目。南充新能源汽车产业园、南充经开区等工业园区加快发展，全市工业园区建成面积达105平方公里。国家现代农业示范区、国家农业科技园充实扩容，中法农业科技园基本建成。南充现代物流园成功跻身国家级示范物流园区。

　　（三）狠抓城乡统筹、生态建设，人居环境展现新面貌。 坚持全域发展理念，统筹推进区域和城乡协调发展。新型城镇化步伐加快。全市城镇化率提高1.4个百分点、达到46.47%。中心城区建成区面积126平方公里、常住人口125万人，清泉坝、下中坝、白马新城等城市新区有序拓展，沿江两岸、城市门户等三区重要节点功能风貌实现全新提升。6个县城发展品质明显改善、亮点迭出，23个“百镇建设试点行动”镇加快建设，613个幸福美丽新村全面建成。城市管理力度不断加大，数字城管作用逐步发挥。污染防治成效明显。全力打好污染防治“三大战役”，主城区PM2.5年均浓度较基准年下降24.6%，空气质量优良天数率达80%、创近4年新高。全面落实河长制，实现市县乡村四级河长、河道警长全覆盖，嘉陵江干流、升钟湖保持Ⅱ类水质，5个国省考核断面全部达标。中心城区、县城污水集中处理率分别提高到88.7%、82.7%，生活垃圾无害化处理率分别提高到95%、85%。按期完成中省环保督察发现问题整改，全面完成992家禁养区内畜禽养殖场关停搬迁；环保部挂牌督办的主城区饮用水源地迁建工程提前完成，困扰多年的主城区饮用水源地环境问题得到解决。生态环境持续改善。嘉陵江流域生态文明先行示范区建设加快推进，成功创建国家级森林公园2个、湿地公园1个，全市森林覆盖率提高到40.06%，人均公园绿地面积达12.03平方米。节能减排任务全面完成，单位GDP能耗下降4.7%。

　　（四）狠抓改革创新、对外开放，经济发展释放新动能。 坚持对内靠改革、对外靠开放，经济活力和韧性明显增强。供给侧结构性改革稳步推进。大力推进“三去一降一补”，淘汰落后产能企业161户；积极推行货币化安置，商品住宅去库存周期降至8.8个月；强化“去杠杆”和债务风险管理，全市政府性债务风险总体可控；全面落实减税降费政策，降低水电气等要素成本7.3亿元。重点改革持续深化。158项中长期规划任务加快推进、213项年度任务圆满完成、36项国省试点加速突破。商事制度改革持续深化，“二十八证合一”全面推行。公立医院综合改革获得国务院通报表彰。创新创业活力迸发。成功创建国家创新驱动助力工程示范市、国家知识产权试点城市。“3+N”创新创业孵化体系基本形成，全市孵化器面积达70万平方米。新增科技型企业567户、国家高新技术企业13户、市场主体4.2万户。14项科技成果获国省科技进步奖，总量居全省第3位；发明专利申请量增长74%，科技对经济增长贡献率达48%。开放水平大幅提升。精心举办“2017中国知名企业南充行”等重大投资促进活动8场，全年新签约项目208个，实现到位资金580.6亿元、增长20.9%；投资200亿元的中铁联运等8个“百亿级”项目成功签约，创造了南充招商史上多项之最。全市外贸进出口总额达19.6亿元、增长81.9%。

　　（五）狠抓脱贫攻坚、民生改善，人民生活实现新跃升。 坚持严守底线民生、改善重点民生、保障基本民生，努力使改革发展成果更好惠及全市人民。脱贫攻坚连战连捷。南部在全国首批国贫县考核验收中高分通过、名列前茅，蓬安在全省省贫县考核验收中排位靠前、成功出列，仪陇、嘉陵、高坪脱贫摘帽顺利完成省级验收评估，全市468个贫困村成功退出、10.7万人实现脱贫。建成脱贫奔康产业园876个、脱贫示范线3条，带动7.2万贫困户长效增收。完成3.7万人易地扶贫搬迁、2.8万户农村危房改造、748户地灾避险安置，解决9.8万建档立卡贫困人口饮水安全问题。建成县级以上“四好村”1129个。资助贫困家庭学生72.5万人次，县域内贫困人口医疗费用个人支付占比控制在10%以内。贫困家庭劳动力新增转移就业2万余人。民生实事扎实推进。十项民生工程、20件民生实事、135个民生项目全面完成，民生投入占公共财政支出比重达73.5%。营山饮用水工程、市第四自来水厂等重大民生项目加快实施，完成城镇危旧房棚户区改造13482套。建成农村公路1500公里，完成渡改桥23座。覆盖城乡的社会保障体系不断健全，新增城镇就业8.5万人，城镇登记失业率控制在3.88%以内；“五大保险”参保1061.4万人次，社会救助体系更加完善。社会事业加快发展。学前教育、义务教育、高中阶段教育入学（园）率分别为86%、100%、94%，均高于全国全省平均水平。嘉陵江文化旅游职业学院、四川建华职业学院成功列入国省“十三五”高校设置规划。新增医疗床位3970张、养老机构床位3600张，市中心医院入选2017（首届）中国医院百强院。成功举办第二届南充国际木偶艺术周、四川省第二届农民艺术节。社会治理有效加强。深入推进“法治南充”“平安南充”建设，切实强化安全生产监管，扎实抓好防汛减灾和食品药品安全，有效防范和化解各类风险，全市社会大局保持和谐稳定。

　　（六）狠抓职能转变、环境优化，自身建设取得新进展。大力倡导“五种精神抓落实”，进一步展示了新作为、树立了新形象。深入推进法治政府建设。办理市人大代表建议317件、政协委员提案390件，办结率100%。在全省率先建成“1+6”重大行政决策制度体系。法治政府建设在全国100个被测评城市中排第33位、上升53位、居西部17个地级市首位。深入推进诚信政府建设。坚持以政务诚信引领社会诚信，积极倡导契约精神，我市综合信用指数在全国261个被测评地级市中排第92位、上升125位、居全省第4位。深入推进开放政府建设。全面推行决策、执行、管理、服务和结果“五公开”，通过邀请市民代表列席市政府有关会议等方式，不断拓宽政务公开渠道。深入推进效能政府建设。深化“放管服”改革，项目审批办结时限提速79.6%，我市成为川渝两地审批时限最短地区。“12345”政务服务热线成为全国范本。核减“同一人证明”等不合理证明250项，仅保留必要证明14项，有效解决办证多、办事难问题。深入推进廉洁政府建设。常态化制度化推进“两学一做”学习教育，严格落实中央八项规定和实施细则精神、省委省政府十项规定和实施细则精神，全面执行市委市政府九项规定和实施细则，坚决纠正“四风”。加强行政监察和审计监督，强力推进政府系统反腐倡廉，风清气正的良好政治生态持续巩固。

　　与此同时，第十届村（居）委会换届选举全面完成，国防动员、人民防空和双拥优抚工作得到加强，国资监管、检验检疫、统计调查、国土、质监、海关、气象、档案、地方志、保密、国安、信访、邮政、贸促、外事侨务、驻市外机构、港澳台等工作取得新成绩，民族宗教、精神文明、慈善、老龄、妇女儿童、青少年、残疾人、工会、红十字、爱国卫生等事业有了新进步。

　　各位代表！实干筑就梦想，拼搏创造实绩。过去的一年，我们办成了一些难事、干成了许多大事，成绩令人振奋和鼓舞。这些成绩，是省委、省政府和市委坚强领导的结果，是市人大、市政协和人大代表、政协委员监督支持的结果，是全市广大干部群众苦干实干、奋发努力的结果。在此，我代表市人民政府，向全市人民，向各位代表、各位委员，向各民主党派、工商联、无党派人士和各人民团体，向驻市解放军、武警官兵和政法干警，向所有关心和支持南充发展的海内外朋友们表示衷心的感谢和崇高的敬意！

　　我们也清醒认识到，全市经济社会发展仍面临一些问题和不足：经济转型任重道远，工业投资、民间投资占比仍然偏低，新兴产业发展不足；脱贫摘帽仍在路上，民生保障面宽量大，公共服务供给离群众期盼还有一定差距，城市精细化管理水平有待进一步提高；金融、环保、安全生产等领域风险防范压力较大；政府职能转变仍需深化，一些公职人员的能力本领、实干作风有待进一步加强。对此，我们将本着对党和人民高度负责的态度，直面问题、勇于担当，采取有力举措，认真加以解决。

二、2018年工作安排

　　各位代表！奋斗不息，梦想可期。中国特色社会主义进入新时代，南充发展站上新起点。市委六届七次全会对实现“南充新未来·成渝第二城”战略目标进一步作出了“三步走”的战略部署，这是跨进新时代乘势而上的科学抉择，也是站在新起点勇开新局的责任担当，使南充发展的宏伟蓝图变得更为清晰，让全市人民的奋斗目标变得更加具体。我们一定要不忘初心、满怀信心、坚定决心，坚定不移向着“南充新未来·成渝第二城”阔步前进！适应新时代、聚焦新目标、落实新部署，要求我们必须牢牢把握习近平新时代中国特色社会主义思想，牢牢把握以人民为中心的发展思想，牢牢把握市委“三步走”战略部署，加速壮大“五大千亿产业集群”、统筹培育“五大百亿战略性新兴产业”，强力推进“五大板块重大工程项目”、扎实抓好“100件大事”，以永不懈怠的精神状态和一往无前的奋斗姿态，奋力谱写南充强势突破的新篇章，不断展现南充大城崛起的新精彩！

　　2018年是贯彻党的十九大精神的开局之年，是改革开放40周年，也是加快建设成渝第二城的深化之年，做好政府工作意义特殊、责任重大。今年政府工作的总体要求是：坚持以习近平新时代中国特色社会主义思想为指导，深入贯彻党的十九大和中央经济工作会议精神，全面落实省第十一次党代会、省委十一届二次全会、省委经济工作会和市委六届七次全会、市委经济工作会决策部署，坚持稳中求进工作总基调，坚持高质量发展要求，坚持“项目建设年”部署，聚焦“三稳三进三突破”总目标，按照市委“坚决打赢‘六场攻坚战’，坚决打赢‘三场持久战’，坚决抓好‘五大统筹’”总安排，深入实施“155发展战略”，做实做好稳增长、促改革、调结构、惠民生、防风险各项工作，大力推动质量变革、效率变革、动力变革，促进全市经济社会持续快速健康发展。

　　全年经济社会发展主要预期目标是：地区生产总值增长8%以上，全社会固定资产投资增长12%以上，一般公共预算收入增长8.6%以上，规上工业增加值增长10.1%以上，社会消费品零售总额增长12.7%以上，服务业增加值增长9.8%以上，城镇和农村居民人均可支配收入分别增长8.6%、9.5%以上，城镇化率提高1.4个百分点以上。

　　围绕上述目标，今年重点要抓好九个方面的工作。

　　（一）加快推动产业集群发展

　　加速壮大汽车汽配产业。开工建设吉利二期、重庆银翔等项目，加快推进博萨汽配、欧度电动车等项目，确保中国重汽、顺发机械、立民制冷等项目竣工投产。不断提升产业配套协作水平，引进重点汽车汽配生产企业6户以上。大力推广运用“南充造”新能源汽车，新建公共充电设施240处以上，增开纯电动公交专线2条以上。确保汽车汽配产业产值增长16.1%以上、突破700亿元。

　　加速壮大油气化工产业。加快建设蜀能工业异辛烷、顺城盐化等项目，推动晟达PTA项目投产达效，力促青龙化工、鑫安邦定、蜀爱新材料等项目建成投产；推动混合芳烃等项目早日落地。大力开展沿链招商，招引重大油气化工项目3个以上。积极推进智能工厂试点示范，加快智慧化工园区建设。确保油气化工产业产值增长25%以上、达到350亿元。

　　加速壮大丝纺服装产业。积极推进蚕桑基地建设。加快丝绸产业技术集成创新，新增省级技术创新研发中心2个以上。推动新丝路国际生态文旅城等项目开工建设，确保东达立新、银河地毯等技改项目竣工投产。深入挖掘南充丝绸品牌价值，制定南充丝纺服装产品地方质量标准，争创中国质量奖、地理标志产品等品牌6个以上。积极创建中国丝绸文化名城。确保丝纺服装产业产值增长11.8%以上、达到445亿元。

　　加速壮大现代物流产业。加快建设川东北粮食物流中心等项目，确保中国物流南充分中心、万里伟业物流中心等项目全面建成。支持浙江传化、南鑫国际、友信崧锋等企业做大做强。引进物流项目15个以上，创建国家A级物流企业3家以上，新增城乡配送网点400个以上，确保10家以上品牌快递区域总部进驻南充。推动6个县级物流节点建设，持续完善市域物流体系。确保现代物流产业产值增长16.6%以上、达到455亿元。

　　加速壮大现代农业。持续深化农业供给侧结构性改革，新建循环农业园区20个以上、新业态示范样板20个以上，培育产值过亿元龙头企业20家以上。打响“南充嘉作”等农产品区域品牌，新认证“三品一标”农产品30个以上、有机农产品基地5万亩以上。扎实推进亭子口灌区工程前期工作，加快建设升钟灌区二期、营山金鸡沟水库；新增（恢复、改善）有效灌面40万亩、高标准农田30万亩。认真落实粮食安全省长责任制部署要求，坚决保障粮食安全。确保现代农业产业产值增长10.2%以上、达到1020亿元，提前实现千亿目标。

　　加速壮大“五大百亿战略性新兴产业”。大力培育电子信息百亿产业。推动三环六期加快建设、七期竣工投产，启动建设暴风启智、鑫派电子等项目；积极承接成渝集成电路及电子器件、新型显示、人工智能等产业，着力打造成渝电子信息产业重要配套区；加快建设南充云计算中心和大数据服务平台，着力打造区域通讯信息枢纽中心。大力培育新材料百亿产业。积极发展新型功能材料、先进结构材料、高性能复合材料等产业，开工建设中远新材料、中城建新型建筑材料等项目，力促鑫达二期等项目竣工投产，着力打造省内一流、西部领先的新材料生产研发基地。大力培育高端装备制造百亿产业。重点发展真空设备制造、特种装备制造、精密智能制造产业，培育壮大九天真空、驭云航空等企业，推动中铁联运智能化托盘加工基地加快建设、新页科技等项目签约落地，着力打造全省重要的高端装备制造基地。大力培育生物医药百亿产业。依托太极制药、香雪制药、康美药业等企业，加快建设营山川芎、南部白芨等道地中药材基地，大力发展化学新药、现代中药等生产技术和产品，着力打造中国西部医药产业园、中国西部生物医药产学研基地。大力培育节能环保百亿产业。围绕高效节能、先进环保、资源循环利用等领域，推广应用大气污染防治、污水处理与废水资源化、固体废弃物处理、建筑节能等环保技术，积极开发高性能脱硫剂、固废处理固化剂等环保产品，加快建设市医废处理中心扩能、危废处理中心、南部国能生物等项目，推动一批节能环保项目竣工投产。

　　（二）强力推进重点板块项目

　　强力推进城市建设重大项目。实施重大项目183个，完成年度投资558亿元以上。启动阳春路等城市道路标美化提升，开工建设将军路嘉陵江大桥、南部嘉陵江三桥等项目，大力实施西山城市公园、北湖片区改造提升工程，加快建设中央森林公园、双女石公园等一批城市公园，大力完善高铁商务区基础设施，确保上中坝“印象嘉陵江”等项目全面完工，滨江中路互通竣工通车。建成地下综合管廊15公里以上。开展新一轮城市总体规划修编，完成城市快速路网等规划编制。

　　强力推进交通枢纽重大项目。实施重大项目137个，完成年度投资140亿元以上。力争蓉京高铁成南达段、汉巴南铁路启动建设，完成高铁站选址。全面完成“南充交通三年推进计划”，开工建设南潼、阆仪营、南充二绕等高速公路，加快建设营达高速公路，确保绵南高速公路建成通车。启动建设顺仪、西顺嘉等一级公路，加快推进顺蓬营等干线公路，确保定升公路、都京港嘉陵江大桥建成通车。完成高坪机场航站楼改扩建，新增航线3条以上，开工建设阆中机场。建成都京港作业区物流中心、嘉陵江航运配套二期。

　　强力推进教科文卫重大项目。实施重大项目53个，完成年度投资170亿元以上。加快建设嘉陵江文化旅游职业学院、四川建华职业学院，启动市体育中心迁建，大力推进南充职业技术学院新区二期等项目，新（改、扩）建校舍、运动场70万平方米以上。加快推进南充高新孵化园二期等重点孵化器建设，完成吉利新能源汽车产业技术研究院组建，推动三环研究院开工建设。启动建设农耕文化博物馆，加快推进市博物馆、市文化馆等项目，完成南充大剧院升级改造。加快市中心医院江东医院、仪陇县人民医院新院区等项目建设。

　　强力推进旅游三产重大项目。实施重大项目22个，完成年度投资100亿元以上。大力实施旅游“双百亿工程”，重点推进阆中水城、嘉陵天乐谷等项目，加快推动顺庆西河生态乡村旅游开发、蓬安相如故城保护开发、朱德故里景区红色旅游融合发展，确保旅游总收入增长20%以上、突破500亿元。深入推进服务业“三百工程”，启动建设国际会展中心等项目。继续实施“全企入网、全民触网、电商示范”，大力推进全国电子商务进农村综合示范项目建设。加快建设川东北金融中心，启动金融商业楼宇及配套工程建设，新入驻金融和准金融机构50家以上。

　　强力推进产业园区重大项目。实施重大项目89个，完成年度投资180亿元以上。加快建设南充新能源汽车产业园，着力引进一批领军型补链型企业；大力抓好南充经开区发展规划修编，争创国家级经开区；支持县（市、区）工业园区加快发展。有序拓展国家现代农业示范区产业基地规模，加快推进川东北农产品精深加工园区建设；大力推动国家农业科技园做强核心区、提升示范区、激活辐射区，确保通过国家验收；推动中法农业科技园全面建成开园。大力实施南充现代物流园三年行动计划，用好国家级示范物流园区“金字招牌”。

　　强力推进“100件大事”。市委六届七次全会谋划推出的“100件大事”，是中央大政方针、省委省政府战略安排和市委决策部署的具体化、工程化、项目化，是对“五大板块重大工程项目”战略抓手的丰富和完善。我们将坚持把“100件大事”作为牵引带动全局工作的“牛鼻子”，扭住不放、一抓到底，挂出作战图、列出任务书、倒排时间表，以务实的举措把每件大事抓实抓好，让群众真真切切感受到大城崛起的强劲态势，实实在在看到南充发展的可喜变化。

　　（三）坚决打赢脱贫攻坚硬仗

　　聚焦目标精准发力。坚持尽锐出战、精准施策，紧扣“两不愁、三保障”“四个好”目标，按照“六个精准”要求和“五个一批”部署，深入开展精准脱贫“千日攻坚”行动，全面落实24个扶贫专项年度计划，坚决实现阆中和营山摘帽、411个贫困村退出、7.8万贫困人口脱贫、全市基本脱贫“3+1”目标。切实用好“六有”管理平台，进一步提高贫困识别和退出精准度，坚决防止数字脱贫、虚假脱贫、形式脱贫。

　　聚焦短板精准发力。扎实抓好住房安全工作，完成易地扶贫搬迁2.9万人、农村危房改造2万户、地灾避险安置500户。继续加大对贫困家庭学生资助力度，确保适龄儿童义务教育不辍一人。全面推行“先诊疗、后付费”就医模式，落实“十免四补助”政策，完成贫困村卫生室和所在乡镇卫生院达标建设。加快补齐贫困地区交通、水利、电力、通讯等基础设施短板，切实改善群众生产生活条件。用好用活“四项基金”，建立稳定补充机制，保持合理基金规模，力争发挥更好作用。

　　聚焦长效精准发力。突出抓好产业扶贫，新建脱贫奔康产业园496个以上，发展特色产业基地30万亩以上，巩固提升3条脱贫示范线，建成脱贫示范村125个以上。全面实施就业扶贫“四千工程”，确保贫困家庭劳动力新增转移就业1万人以上。扎实开展精准脱贫“三大行动”“五项教育”，引导群众养成好习惯、形成好风气。强化“回头看”“回头帮”，科学设置贫困监测点，对可能返贫对象提前预警、提前介入，确保长效脱贫、稳定增收。

　　（四）深入推进环境污染防治

　　解决突出环境问题。打好蓝天保卫战，扎实抓好大气污染联防联控联治，强力整治城市扬尘、露天焚烧、餐饮油烟、汽车尾气、工业废气，确保主城区PM2.5年均浓度较基准年下降24.6%以上。打好碧水保卫战，充分发挥河（湖）长制、河道警长制作用，扎实开展重点流域水污染综合治理攻坚行动，全面完成县城饮用水源安全隐患整治任务，切实增强场镇污水收集处理能力，确保国省考核断面水质优良比例达100%。打好净土保卫战，全面开展土壤污染状况详查，大力实施土壤污染治理与修复试点示范工程，确保固体废物、重点工业污染和农村面源污染治理取得实效。统筹推进中省环保督察发现问题整改，坚决做到对标完成、按期销号。

　　大力加强生态建设。扎实抓好嘉陵江流域生态文明先行示范区、嘉陵江（南充段）绿色生态走廊建设，加快创建国家生态市。支持仪陇创建国家园林县城、蓬安创建省级园林县城，加快南部升钟湖、营山清水湖、蓬安相如湖等国家级湿地公园（试点）建设。加大天然林保护力度，完成退耕还林还草3万亩以上。深入开展大规模绿化南充行动，确保全市森林覆盖率达到40.41%。

　　着力推进绿色发展。树立和践行绿水青山就是金山银山的理念，实施循环发展引领行动计划，启动建设顺高嘉、阆南西、仪营蓬三大片区固废循环经济产业园。加快建设国家餐厨垃圾资源化利用和无害化处理示范市，建成餐厨废弃物处理项目。开展节约型机关、绿色社区等创建活动，倡导绿色低碳生产生活方式。大力推进节能降耗，单位GDP能耗下降3.5%以上。实施领导干部自然资源资产离任审计，实行生态环境损害责任终身追究，建立生态环境损害赔偿制度，坚决守护好南充的绿水青山。

　　（五）有效防范化解重大风险

　　防控金融风险。完善地方金融监管体系，促进地方金融机构健康发展。健全企业债券违约处置机制，积极推进市场化法治化债转股，着力化解企业债务风险。强力查处金融乱象，坚决打击非法集资等违法违规金融活动，稳妥有序推进互联网金融风险专项整治，鼓励银行业机构妥善处置不良资产。规范地方政府举债行为，建立政府性债务全口径监管机制，严禁违法违规举债担保和变相举债，有效遏制债务增量、有序化解债务存量。

　　防控安全风险。严格落实安全生产责任制，强化安全生产防控和隐患治理体系建设。大力实施安全生产“三大行动”“五大工程”，深入开展重点行业领域大排查、大整治，强力推进“打非治违”，坚决遏制重特大安全生产事故发生。加大食品药品质量监督抽查力度，加快市食药检验检测中心、智慧食药监管平台建设，积极创建食品安全示范城市。着力提高防灾减灾救灾能力，扎实抓好防汛抗旱、地灾防治等工作。

　　防控稳定风险。加强社会稳定风险评估，完善矛盾纠纷多元化解机制，从源头上防范化解各类矛盾纠纷。深入推进涉法涉诉问题与信访分离，严格执行处理、复查、复核信访三级终结制度，及时解决群众合理合法诉求。深入推进“平安南充”建设，加快完善立体化、信息化社会治安防控体系，强力开展扫黑除恶专项斗争，大力开展反渗透、反分裂、反恐怖、反邪教斗争，坚决打击网络谣言和电信诈骗，全力维护全市和谐稳定大局。

　　（六）积极扩大对外开放合作

　　全方位融入国家开放战略。深度融入“一带一路”建设，务实开展与俄罗斯、法国、德国等国家友好城市交流合作，积极吸引国际投资基金、海外资本、高端人才、先进技术、优质项目进驻南充。大力实施“走出去”战略，积极参加“万企出国门”“千企行丝路”等活动，引导企业参与国际竞争、拓展海外市场。启动高坪机场申报航空口岸前期工作，推动设立保税物流中心（B型），确保南充公共保税仓投入运营。

　　宽领域推进区域合作发展。积极融入长江经济带发展，拓展与京津冀、长三角、珠三角等重点区域的对接交流，加强与成渝城市群、川陕革命老区、川渝陕甘结合部城市对接，努力在更大范围、更高层次配置资源。全面落实省委川渝合作发展要求，积极对接两江新区、天府新区，深度融入四川、重庆自由贸易试验区，做强重庆配套产业园，争取设立四川自贸区南充协同改革开放园区。

　　多层次开展投资促进活动。坚持招大引强、招强引优，深入实施“招商大会战”。积极参加“西博会”“广交会”“中外知名企业四川行”等重大招商活动，精心举办五大千亿产业集群专题投资推介等“十大招商活动”。发挥驻市外机构“前哨”作用，构建辐射全国的招商网络。确保全年招引5亿元以上产业项目100个、到位资金增长100亿元以上。

（七）持续深化全面创新改革

　　深化供给侧结构性改革。重点在“破”“立”“降”上下功夫，大力破除无效供给，着力处置一批“僵尸企业”，推动化解过剩产能；采取有效举措降低非住宅商品房库存，促进房地产市场平稳健康发展。加快建设质量强市，大力推进品牌建设，新增注册商标1000件以上，争创中国驰名商标等品牌10个以上。扎实开展降成本行动，清理规范涉企收费，落实降低制造业增值税、社会保险费等政策，切实减轻企业负担。健全补短板协作机制，吸引更多社会资本参与交通、环保等基础设施建设。

　　有序推进重点领域改革。大力推进地方金融改革，加快蓬安农信联社改制步伐。深化财税制度改革，改进预算管理制度。加快推进国资国企改革，做实做强“1+5”市级投融资公司。深化实施公立医院综合改革，全面推进医疗医保医药“三医”联动改革，确保县域内就诊率稳定在90%以上。扎实抓好经济体制、生态文明、农业农村等重点改革，加快推进阆中国家新型城镇化、南部国家中小城市综合改革等国省改革试点，积极争取一批新的国省改革试点，努力走出具有南充特色的改革路子。

　　充分激发创新创业活力。加快建设国家创新驱动助力工程示范市、国家知识产权试点城市，积极争创国家高新技术产业开发区、川东北军民融合创新产业园。深入实施“嘉陵江英才工程”，全面实施科技型中小微企业“奔腾计划”、专利倍增“奔涌计划”、创客南充“奔云计划”，加强知识产权保护，力争引进高层次创新创业人才200名以上、团队5个以上，新增科技型企业1500家以上、市场主体2.1万户以上，申请国家发明专利600件以上。实施重大科技创新项目60个以上。

　　（八）大力实施乡村振兴战略

　　夯实产业基础。坚持全域布局规模化发展、区域布局差异化发展、流域布局特色化发展，加快构建现代农业产业体系、生产体系、经营体系。大力发展晚熟柑桔等特色产业。推动农业与乡村旅游、健康养老等多种业态深度融合，乡村旅游收入增长15.3%以上。大力发展多种形式适度规模经营，新增农民合作社100个、家庭农场300家、农民职业经理人120名、新型职业农民3000人以上。

　　抓好新村建设。大力实施农村人居环境整治行动，启动“果州新家园”试点，建成幸福美丽新村500个，改造（拆除）土坯房3万户以上。加快完善基础设施，新建通社通户道路2200公里，巩固提升38.4万农村人口饮水安全，完成172个村农网改造升级。建成“1+6”村级公共服务中心240个以上。统筹推进山水林田湖草系统治理，建成150个村庄垃圾收集转运处理设施，全面改善乡村人居环境。

　　培育乡风文明。大力推进乡村文化振兴，创建县级以上“四好村”1200个。深入实施乡村公民道德“百人千村”宣讲计划，常态化开展“六顺六净”行动，推动乡村移风易俗、文明进步。开展好“千名农技能人”评选、文明村、文明户创建活动，建成县级以上文明村镇850个以上、文明家庭1000个以上。

　　完善乡村治理。健全自治、法治、德治相结合的乡村治理体系。积极扩大基层民主，切实保障群众民主权利。创新乡村依法治理机制，推行村级组织“小微权力清单”，实现村（社区）法律顾问全覆盖。完善村规民约，创新乡贤文化，弘扬善行义举。扎实推进平安乡镇（村庄）建设，扩大“雪亮工程”覆盖面。强化乡村振兴人才支撑，打造一支懂农业、爱农村、爱农民的“三农”工作队伍。

　　促进群众增收。鼓励支持农民就业创业、拓展增收渠道，确保全市转移劳动力稳定在220万人以上。健全农村产权流转交易体系，抓好农村产权抵押融资试点。巩固和完善农村基本经营制度，深化农村土地制度改革，全市30%的行政村成立土地股份合作社；推进承包地“三权”分置并行，引导土地流转165万亩以上。完成全市集体资产清产核资，不断壮大村级集体经济实力。

　　（九）全力增进人民群众福祉

　　切实办好民生实事。持续加大民生投入，确保民生投入占公共财政支出比重稳定在70%以上。大力实施十项民生工程和20件民生实事。抓好高校毕业生、农民工等重点人群就业工作，确保城镇登记失业率控制在4.2%以内。建成市第四自来水厂等一批民生项目，完成城镇危旧房棚户区改造4.5万套，新（改）建农村公路1500公里，实施渡改桥14座。大力推动“厕所革命”。全面实施全民参保计划，“五大保险”参保达到1062万人次。不断完善社会救助体系。扎实推进住房租赁试点，努力让群众住有所居、安居乐业。

　　切实强化公共服务。优先发展教育事业，支持西华师范大学创建博士学位授予单位、西南石油大学（南充）创办高水平应用型本科院校、川北医学院创办高水平医科大学；大力推进城乡义务教育一体化改革发展，继续实施高中阶段教育普及攻坚计划、学前教育行动计划、特殊教育提升计划，持续提升职业教育、民办教育质量，着力解决中小学生课外负担重、“择校热”“大班额”等问题。广泛开展文化惠民活动，精心筹办第六届嘉陵江合唱艺术节。加快推进健康南充建设，启动创建国家中医药先进市。积极创建省级文明城市。

　　切实加强社会治理。推动政府治理、社会自我调节、居民自治良性互动。鼓励和引导企事业单位、社会组织、人民群众广泛参与，推动社会组织明确权责、发挥作用。推进社区矫正规范化建设，加强流动人口和特殊人群管理。深化“法治南充”建设，扎实开展“七五普法”、法律“七进”和基层法治示范创建，进一步在全社会形成法治良序。

三、加强政府自身建设

　　各位代表！新时代新使命，新征程新作为。新的一年，我们将深入贯彻习近平新时代中国特色社会主义思想，坚持把政治建设放在政府自身建设的首位，把党的领导贯穿政府工作全过程各方面，进一步牢固树立“四个意识”、坚定“四个自信”，在市委坚强领导下，全面加强政府系统党的建设，认真开展“不忘初心、牢记使命”主题教育，常态化制度化推进“两学一做”学习教育，着力建设人民满意政府。

　　（一）坚持依法行政，着力规范权力运行。严格遵守宪法和法律，始终用法治思维、法治方式履行职责，进一步提高政府工作法治化水平。自觉接受市人大及其常委会的法律监督、工作监督和市政协的民主监督，诚恳接受社会监督、舆论监督和司法监督，让权力始终在阳光下运行。严格执行重大行政决策法定程序，始终做到科学决策、民主决策、依法决策。持续开展法治政府建设第三方评估，做好政府立法、行政应诉工作，加强行政复议规范化建设。深化行政执法体制改革，改进和加强行政执法工作，做到严格规范公正文明执法。

　　（二）坚持重信守诺，切实加强诚信建设。充分发挥政府诚实守信的表率作用，完善政府守信践诺机制，建立健全政务和行政承诺考核制度，大力整治群众反映强烈的政务失信行为。强力推进社会信用体系建设，大力开展“三诚信”提升行动，基本建成覆盖全市的公共信用信息平台。健全守信联合激励和失信联合惩戒机制，促进信用信息广泛有效应用。强化诚信教育和宣传，让诚信成为引领南充发展的精神风尚。

　　（三）坚持开明开放，畅通政群互动渠道。持续抓好权力清单、责任清单、负面清单公开工作。深入开展政务公开标准化、规范化建设，建立健全重大决策预公开制度。加强政务公开载体建设，进一步发挥“12345”政务服务热线作用，扩大公众参与渠道和范围。深入推进“互联网+政务服务”，加快对接融入全省一体化政务服务平台，促进实体与网上政务大厅融合服务。

　　（四）坚持务实高效，不断优化政务环境。深化“放管服”改革，强化“双随机一公开”监管。着力构建亲清新型政商关系，强力整治不作为、慢作为、乱作为。健全重大项目、重点工作跟踪落实和责任追究机制，确保各项任务落到实处。加强政府公职人员能力建设，不断提高综合素质和依法履职能力。大兴调查研究之风，促进在一线了解情况、解决问题。积极落实正向激励和容错纠错机制，让广大干部愿干事、敢干事、干成事。

　　（五）坚持从严从实，持续深化廉政建设。 严格执行全面从严治党各项规定，认真落实党风廉政建设责任制，切实加强政府系统反腐倡廉工作。强力整治“四风”问题，密切关注新表现新动向，在反对形式主义、官僚主义上下更大功夫，坚决防止反弹回潮。规范开展重点廉政风险岗位抽查审计，不断完善廉政风险防控措施，铲除滋生腐败的土壤，进一步树立良好政务形象、巩固良好政治生态。

　　各位代表！百舸争流，破浪者领航；千帆共进，奋勇者当先。让我们更加紧密地团结在以习近平同志为核心的党中央周围，在省委、省政府和市委的坚强领导下，紧紧依靠和团结全市人民，不忘初心、牢记使命，攻坚克难、拼搏实干，为开创“南充新未来·成渝第二城”崭新局面、决胜全面建成小康社会而不懈奋斗！
